# Morphological evolution of creek networks in 10 restored coastal wetlands in the UK

**DOI:** 10.1038/s41597-022-01199-4

**Published:** 2022-04-01

**Authors:** C. Chirol, I. D. Haigh, N. Pontee, C. E. L Thompson, S. L. Gallop

**Affiliations:** 1 LSE, University of Lorraine, Nancy, 54000 France; 2 Ocean and Earth Sciences, National Oceanography Centre Southampton, University of Southampton, Southampton, SO14 3ZH UK; 3 Jacobs, One Glass Wharf, The West Wing, Bristol, BS2 0EL UK; 4 Channel Coastal Observatory, National Oceanography Centre, Southampton, SO14 3ZH UK; 5 School of Science, University of Waikato, Tauranga, 3110 New Zealand; 6 Environmental Research Institute, University of Waikato, Hamilton, 3240 New Zealand

**Keywords:** Geomorphology, Environmental impact

## Abstract

Coastal wetlands provide crucial ecosystem services including flood protection and carbon storage, but are being lost rapidly worldwide to the combined effects of sea-level rise, erosion and coastal urbanisation. Managed Realignment (MR) aims to mitigate for these losses by restoring reclaimed land to tidal influence. Data of creek evolution is critical to assess the performance of design strategies and improve design and implementation practices. This data descriptor provides a dataset of the horizontal morphological evolution of creek systems from various initial conditions in 10 MR schemes across the UK. Using a semi-automated workflow, morphological creek parameters were extracted from 52 lidar datasets at 1 m horizontal resolution spanning 2 to 20 years post-breach. This constitutes the most comprehensive systematic monitoring of MR creek morphology to date. The dataset will assist future MR design and provide baseline morphological information for ecological and biogeochemical surveying.

## Abstract (summary)

**Table:** 

Measurement(s)	Tidal channel morphometry
Technology Type(s)	Lidar
Factor Type(s)	Tidal range
Sample Characteristic - Environment	Coastal wetland • Managed realignment schemes
Sample Characteristic - Location	United Kingdom

## Background & Summary

**Fig. 1:**
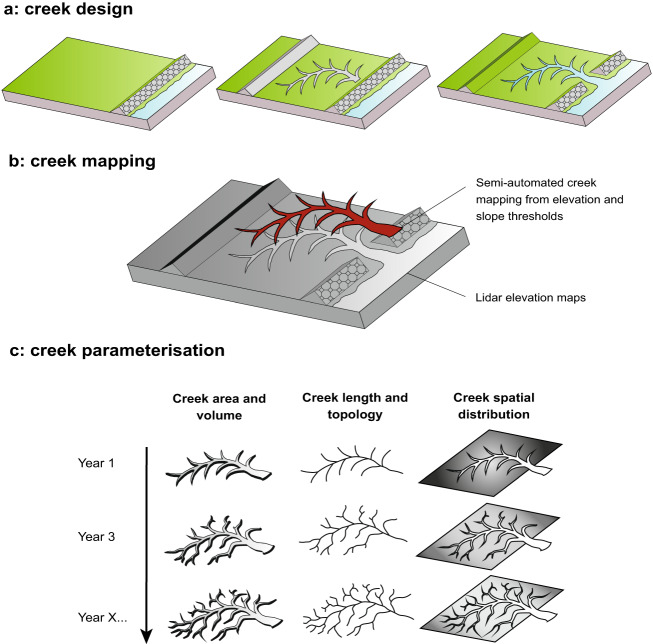
Post-implementation monitoring schemes of creek networks in managed realignment schemes as provided by this data descriptor. (**a**) Initial creek pattern following construction; (**b**) Semi-automated creek mapping from lidar using elevation and slope thresholds; (**c**) Creek parametrisation and morphological evolution of creek systems inferred from consecutive lidar datasets.

Increasing focus on nature-based solutions has led to a rise in coastal wetland restoration projects worldwide^1–3^, to the point where the UN has declared 2021–2030 the Decade of Restoration. Coastal wetlands are being degraded at a rapid pace^4–6^, resulting in the loss of critical ecosystem benefits including biodiversity^7^, flood protection^8^, nursery habitats for juvenile fish^9^, pollutant filtering^10^, and carbon storage^9^. Managed Realignment (MR) schemes, in which embanked lands are opened to tidal influence to create new saltmarsh and mudflat habitats (Fig. 1a), are important sources of experimentation and innovation for coastal restoration strategies and have brought to light design aspects that require further scientific guidance^11–13^. Notably, creek networks play a crucial role in the distribution of water, sediment, nutrients and seeds through the marsh^14–16^, and it is thought that excavating initial channels may speed up creek network development, thus helping to support saltmarsh functioning and meet target conditions^11,17–19^. Of particular interest is the capacity of the initial morphology of MR creeks to develop horizontally and improve their distribution over the marsh.

However, even though a large variety of initial creek designs have been used in the UK and around the world^11,20^, integrated, standardised data sets over time for multiple sites are lacking if we are to compare the impact on creek evolution of different design strategies. This is mainly due to the computational challenge of mapping and parametrising complex features like creek networks. This data gap hinders our understanding of how both the physical and ecological aspects of MR schemes evolve, and hence the formulation of general design and implementation guidelines.

In this data descriptor, we apply a creek mapping algorithm^21^ to 10 MR sites to monitor creek network evolution after implementation (Fig. 1c). The algorithm uses freely-available elevation maps collected from an airborne sensor (lidar) to extract relevant volumetric and topological information (Fig. 1b), and allows for the systematic comparison of non-engineered and highly engineered MR sites. The algorithm was tested on 13 natural saltmarshes that have been morphologically stable for over 20 years, where stability was defined as when geomorphic changes within the creek network within a time period are lower than the data resolution, and so are considered to have reached a mature state^21^. The results were consistent with those found by manual mapping and ground-truthing in the field.

The new dataset provides crucial information on the baseline (pre-breach) morphology of coastal wetland restoration schemes, and how they might evolve in the future. The outputs of this study are twofold: first, topographic maps showing the extent and topology of the creek network for reuse in further studies; second, result tables of creek network morphometric parameters and a series of interpretative figures. The dataset will assist future managed realignment design by showing how current creek systems have evolved since implementation for different environmental conditions and levels of engineering, as well as providing baseline morphological data for further research. Knowledge of the morphology and spatial distribution of creeks is particularly relevant for ecological or biogeochemical surveys. This dataset will therefore support the study of various saltmarsh processes such as headward erosion^22^, seed dispersal^23^, development of vegetation patches^24^, tidal flow and sediment transport^25^, marsh flow attenuation^26^, and carbon storage^27^, and facilitate the interpretation of local observations at the scale of the entire saltmarsh. There is a strong need to conduct such surveys because current MR schemes often fail to provide the same ecosystem services as their natural counterparts^28,29^, and do not replicate the full plant diversity of natural wetlands^30^. If not addressed properly, this could lower their value as wetland loss mitigation schemes.

## Methods

The extraction of MR creek evolution datasets involved three main stages, explained in the details below:

### Stage 1: Site selection and raw data acquisition

The first stage consisted of collating raw datasets that provide geomorphological and tidal information for MR sites, as well as background information on the context and design choices of these MR sites to inform site selection.

### Lidar data

Lidar is an airborne sensor that measures distances by transmitting a laser pulse and calculating the return time of the reflected beam. Lidar is particularly efficient for the geomorphological monitoring of coastal environments^31,32^. The use of lidar is increasing due to its accessibility in many countries, high coverage (e.g., all of the UK territory), high horizontal resolution (up to 0.25 m horizontally), and the possibility to compare datasets over several years to infer evolution rates.

The lidar data used in this data descriptor were collected by the Environment Agency (EA) and accessed via their data services platform^33^. The datasets were interpolated from a point cloud into a raster using a nearest neighbour interpolation to Euclidean distance method. Contrary to other interpolation methods, nearest neighbor preserves variations in the data such as small (~1 m) channels which would otherwise be smoothed over^31^, making this method particularly suited to representing creek networks^34^. The freely available data comes in two forms: a raw Digital Surface Model (DSM) interpolated from the points of first laser beam return, and a Digital Terrain Model (DTM) where the above-ground features such as buildings and vegetation have been filtered out to obtain a bare-Earth model using EA proprietary algorithms^35^.

Horizontal resolutions vary between 0.25 m and 2 m. Due to the fractal behaviour of creek networks^36^, their morphological complexity increases with the horizontal resolution. Thus, in order to compare the morphological evolution of the site at different years, all datasets need to be at the same resolution. The most common available horizontal resolution, 1 m, is therefore used as a standard for this study. The vertical resolution of lidar data is estimated at 0.15 m or lower based on GPS ground-truthing surveys, as described in the EA’s lidar quality control reports^33^.

### Tide data

In order to establish tidal forcing parameters and to estimate the tidal prism, mean predicted tidal levels were obtained for 582 standard (tidal data tabulated) and secondary (tidal data calculated from the standard ports) ports, as provided by the Admiralty Tide Tables 2014^37^. The mean tidal levels at the 13 natural saltmarshes and 10 MR sites were then interpolated from the weighted mean of the surrounding ports’ values (up to 30 km away). Tidal levels were converted from Chart Datum to Ordnance Datum Newlyn, using conversation values in the official Tide Tables and from the National Tidal and Sea Level Facility (https://ntslf.org/). The key tidal levels that were extracted are virtually stationary within the 18.6 year nodal-cycle. Therefore, the effect of sea-level rise was considered negligible in this study which spans a maximum of 20 years of creek evolution. Observed rates of sea-level rise around the English Channel in the 20^th^ century range between 0.8 and 2.3 mm/yr^38^, and have been estimated at 1.4 ± 0.2 mm/yr around the UK^39^, so the change in tidal elevation over 20 years is unlikely to exceed 0.05 m, which lies below the vertical resolution of lidar. The following mean tidal levels are interpolated for each site: Highest Astronomical Tide (HAT), Mean High Water Spring (MHWS), Mean High Water Neap (MHWN), Mean Water Spring (MWS), Mean Low Water Neap (MLWN), and Mean Low Water Spring (MLWS).

### Site selection

**Fig. 2:**
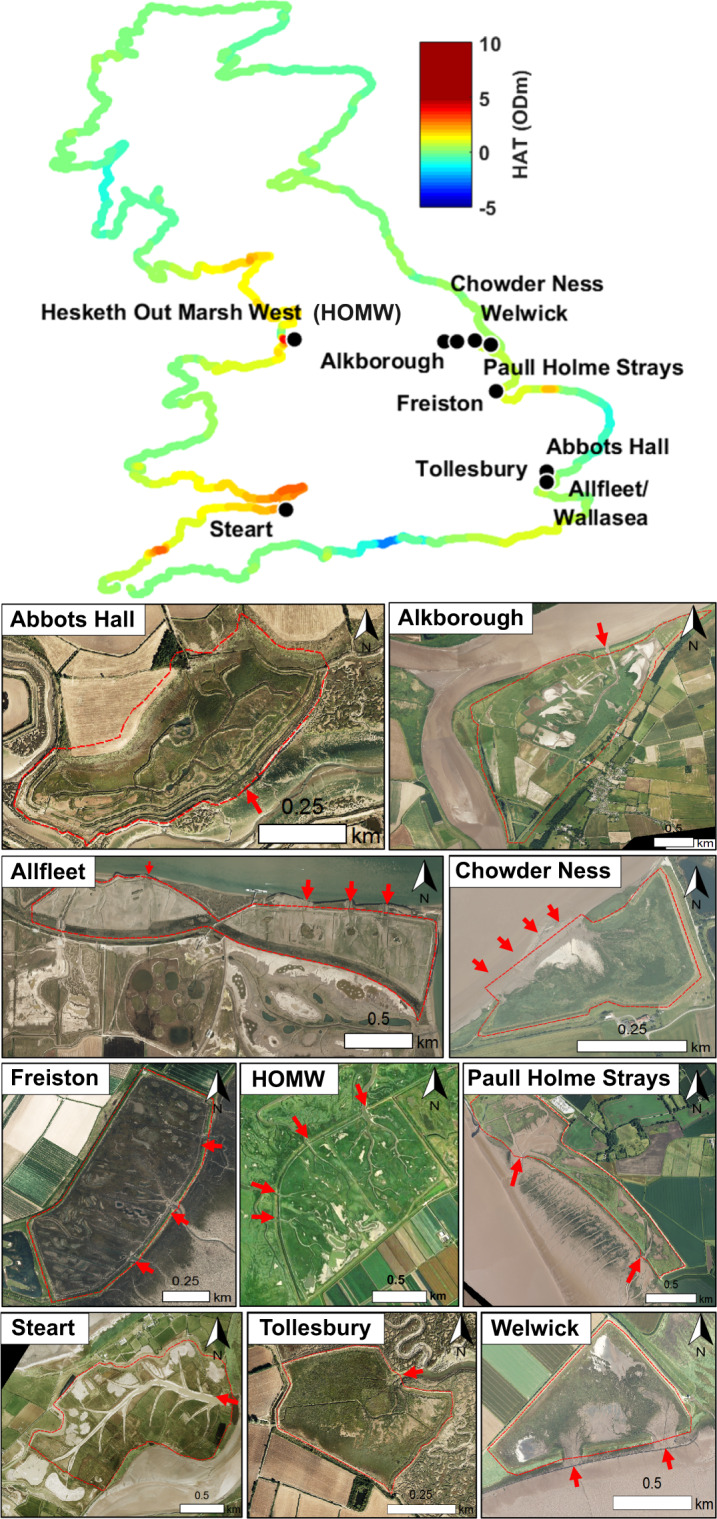
Location and aerial photography of the 10 MR sites considered. HAT: Highest Astronomical Tide interpolated along the British coastline from Admiralty Tide Tables 2014 mean predicted tidal levels. Red dashed lines: catchment area delimited by the remaining seawalls and HAT levels. Red arrows: breach areas.

This study considers 10 MR schemes, implemented between 1995 and 2014 around the coast of England (Fig. 2). Sites were selected based on four criteria the first of which was data availability, i.e. the number of lidar datasets and case studies available. The second criterion was the sites’ settings, which should capture a range of initial external conditions, i.e. the tidal range, size of scheme, location, implementation date, land use history, etc. The third criterion was the general scheme design, i.e. number and size of breaches, initial site elevation within the tidal frame, targeted habitats. The fourth criterion was the creek network design, i.e. the absence of initial creeks (strategy 1), excavation of a creek system from a natural template (strategy 2), or excavation of artificial creeks in the absence of a natural template (strategy 3). The context for each MR scheme was taken from the academic and grey literature (see all references in Table 1), and from an online database referencing coastal habitat creation schemes in North-Western Europe (ABPmer OMReg^40^). The schemes span tidal ranges from 4.1–11 m, granulometries from clay to coarse silt / sand, suspended sediment concentrations from 60–1000 mg/L, mean elevations from 0.9–5.5 m above Mean Water Spring, and scheme areas between 0.1 and 3.61 km^2^.

**Table 1:** Selection criteria and data availability for each site.

	Latitude/ Longitude	Site opening date	EA lidar datasets suitable for use between 1995-2016	Creek design strategy	References
Abbots Hall	52.7846 0.8455	2002	2002; 2008; 2013; 2014; 2015	3	Blott and Pye^57^ Shepherd^58^ ABPmer White Papers^59^ Scott *et al*.^60^ Pendle^61^ Brooks *et al*.^45^
Alkborough	53.6914 −0.6773	2006	2007;2010; 2012; 2015	3	Manson *et al*.^62^ Costa^63^ Medlock *et al*.^64^ Morris^12^ Pendle *et al*.^61^ Luisetti *et al*.^9^ Pontee (in Esteves)^65^ Pontee^11^
Allfleet	52.6163 0.8364	2006	2007; 2011; 2013; 2015	3	ABPmer White Papers^66^ Dixon^67^ Medlock *et al*.^64^ Pendle^61^
Chowder Ness	53.6916 −0.4815	2006	2007; 2009; 2010; 2011; 2012; 2013; 2015; 2016	1	ABPmer White Papers^68^ Luisetti^9^ Costa^63^ Morris^12^ Pendle^61^
Freiston	52.9646 0.0923	2002	2002; 2009; 2011; 2013; 2014	3	Friess^69^ Friess *et al*.^70^ Frost *et al*.^71^ Hampshire^72^ Nottage *et al*.^73^ Rotman^74^ Symonds *et al*.^75^ Symonds *et al*.^76^
Hesketh Out Marsh West (HOMW)	53.7203 −2.8876	2008	2009; 2010; 2011; 2014	2	Hampshire^72^ Pontee (in Esteves)^65^ Tovey *et al*.^77^
Paull Holme Strays	53.7082 −0.2193	2003	2007; 2010; 2012; 2013; 2014	3	Clapp^78^ Costa^63^ Edwards *et al*.^79^ Luisetti^9^ Mazik *et al*.^80^ Morris^12^ Pendle^61^ Pontee *et al*.^81^
Steart	52.1983 −3.0506	2014	2014; 2015; 2016	3	Burgess *et al*.^82^ Townsend^83^ Wright *et al*.^84^
Tollesbury	52.7673 0.8402	1995	2002; 2009; 2012; 2013; 2014; 2015; 2016	3	Atkinson *et al*.^85^ Frost *et al*.^71^ Garbutt *et al*.^86^ Luisetti^9^ Paramor *et al*.^87^ Pendle^61^ Reading *et al*.^88^ Shepherd *et al*.^58^ Steel *et al*.^89^ Watts *et al*.^90^
Welwick	53.6471 0.0095	2006	2007; 2009; 2010; 2011; 2012; 2013; 2014	1	ABPmer White Papers^91^ Costa^63^ Frost *et al*.^71^ Hampshire^72^ Luisetti^9^ Morris^12^ Pontee (in Esteves)^65^ Pontee *et al*.^81^

Creek design strategies considered: 1 = absence of initial creeks; 2 = excavation of a creek system from a natural template; 3 = excavation of artificial creeks in the absence of a natural template.

For each site, we collected 3 to 8 DSMs, surveyed and flown between 2002 and 2016 and spanning 2 to 20 years post-breach depending on data availability. No 1 m resolution lidar dataset could be found before 2002. Furthermore, changes in lidar technology mean that older datasets tend to be noisier. Therefore, while lidar datasets of 2 m horizontal resolution were generally omitted, an exception was made for Allfleet in 2007 in order to quantify the creek network close to the implementation date, and because the data was less noisy than the 2002 1 m resolution dataset taken at Freiston. A summary of selection criteria, lidar data availability and external references is given in Table 1 and the geographical repartition of the selected MR sites is given in Fig. 2.

### Stage 2: Preprocessing

The second stage consisted of a series of preprocessing protocols applied using ArcGIS 10.2.2 to turn the lidar datasets obtained from the EA into the correct format of input files for the creek parametrisation algorithm.

Preprocessing included merging mosaics into a single dataset, interpolating to 1 m horizontal resolution, interpolating data gaps to the values of the nearest neighbours according to Euclidean distance (lidar data are generally collected at low tide when most creeks are drained, but remnant water within ponds for instance may lead to gaps in the dataset), cropping to the saltmarsh area, and extracting elevation and slope maps. The landward limit of the saltmarsh area is defined by the local HAT level which delimits the intertidal zone from the land, and the seaward limit as the mouth of the entry channel, consistent with Steel (1996); both are generally constrained by flood defences in managed realignment schemes. Creek edges tend to be more visible on the slope map than on the curvature map for the selected datasets, so the slope was chosen as a threshold parameter unlike previous studies^41^.

The preprocessing steps are minimalistic to provide monitoring tools that are easily reusable by coastal habitat restoration project designers and researchers for future saltmarsh monitoring efforts. The creek parameters are detected from freely available lidar DSM that have undergone minimal preprocessing, as is likely to be the case for most MR monitoring work performed by environmental agencies, contracted consulting companies, or by research projects in non-geomorphological disciplines that use creek morphology as a baseline. The two outputs of this preprocessing stage, an elevation map and a slope map in degrees, both at a horizontal resolution of 1 m, are converted into text files and exported to Matlab for the processing stage to extract relevant creek morphological parameters.

### Stage 3: Creek mapping, parameters extraction and visualisation

In the third stage, the mean tidal levels and elevation and slope maps obtained in Stage 2 are used as input parameters for a coastal wetland creek parametrisation tool developed in 2014–2018 in collaboration with Jacobs. This tool outputs an Excel table of creek network morphometric characteristics and several figures. The algorithm, written in Matlab R2015a, is made available within the data repository along with the datasets. Readers can also refer a previously published methodology paper^21^ which details the algorithm’s functioning and validation process. A summary of the algorithm’s running principles is given below.

**Fig. 3:**
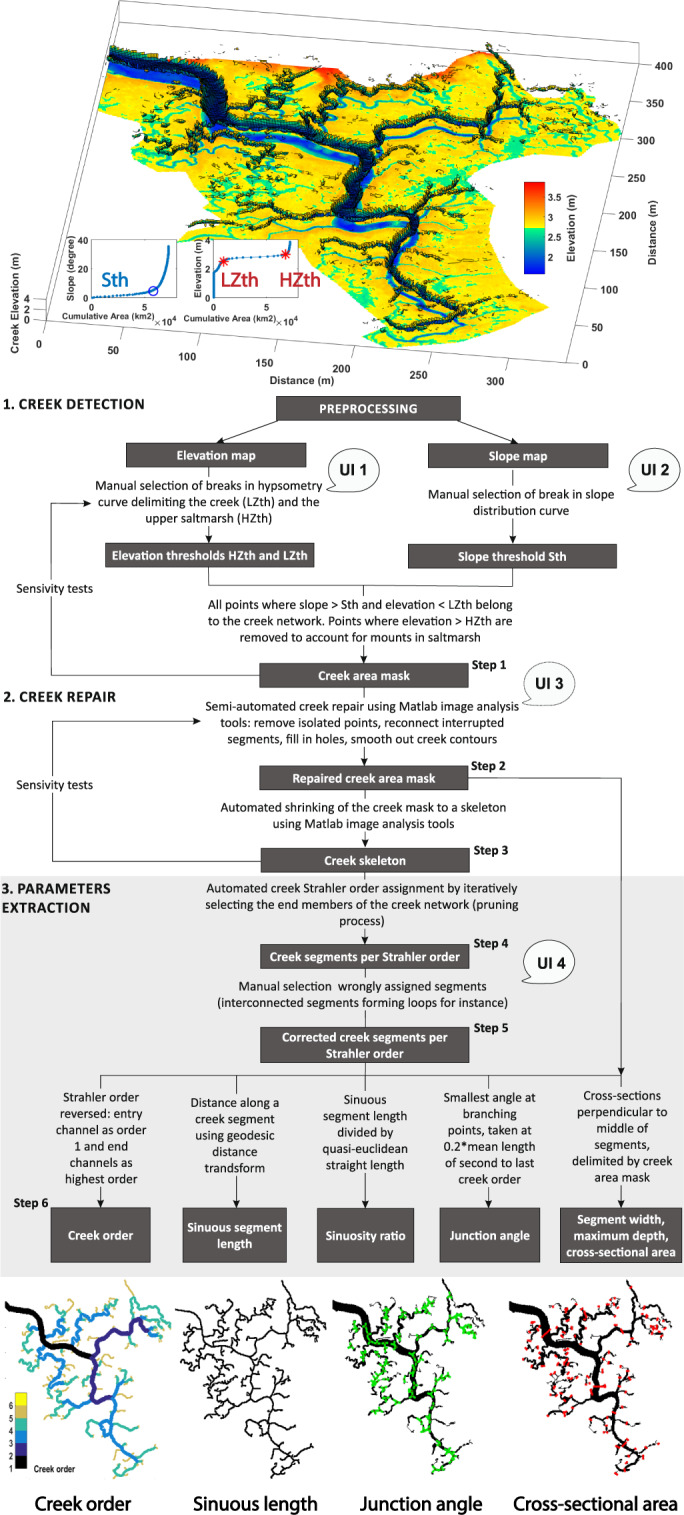
Creek parametrisation algorithm workflow. The 6 processing steps are grouped into three phases: creek detection (step 1); creek repair (steps 2 and 3) and parameter extraction (steps 4 to 6). The steps where user inputs (UIs) are necessary are marked as UI 1 to 4. The algorithm’s functioning and validation process is detailed in a separate publication^21^.

The creek algorithm is based on the threshold method: a creek network is defined as a connected feature which lies lower than the rest of the saltmarsh, and whose edges are delimited by a steeper slope (Fig. 3, Step 1). Once a raw creek logical mask has been detected, noise is filtered out by removing all connected elements smaller than a number of pixels defined by the user, while fragmented terminal channels are reconnected to the creek system using the shortest Euclidean distance as the repair path (Fig. 3, Step 2). Morphological thinning is then applied to shrink the creek network to a skeleton corresponding to the centerline of the channels (Fig. 3, Step 3). The topology of the creek network, expressed quantitatively in terms of reverse Strahler order^21^, is automatically computed by using a pruning process and assigning an order iteratively to all creek segments (Fig. 3, Steps 4 and 5). Compared to traditional Strahler ordering, reverse Strahler order ensures that the entry channel is always classified as the first order. The following parameters are calculated for each creek segment: sinuous length, straight length, sinuosity ratio, junction angle, and cross-sectional width, depth and area (Fig. 3, Step 6). The overall size and distribution of the creek system is given by the drainage density (the total channel length divided by the studied marsh area), overmarsh path length (OPL, the mean distance to the creek system at all points within the marsh), main channel length (the longest channel connected to the largest outlet), total channel length, number of creeks, total mouth cross-sectional area (sum of all outlets’ cross-sectional areas), main channel mouth depth, planform area, creek volume, sinuosity ratio and the main channel gradient.

The algorithm is faster and less subjective than manual mapping, and interactive interfaces make it easy to use by researchers and stakeholders. Furthermore, the short running time at the pixel size used for the study allows for a number of sensitivity tests to refine the threshold parameters. The algorithm therefore allows the extraction of a systematic dataset that is comparable and can be produced quickly across multiple sites and years. Finally, it is well suited for the comparison of creek evolution in non-engineered and highly engineered sites, where creeks shaped by human intervention are poorly detected by the flow accumulation method^42^. A summary of the data types and processing protocols used in this study is provided in Table 2. The algorithm gives as outputs a number of morphometric parameters for the creek network (Table 3), following previous recommendation guidelines for creek design^43^.

**Table 2:** Summary of procedures for each data type.

Type	Lidar DSM	Tide data
**Source**	**Environment Agency**	**Admiralty Tide Tables 2014**
Preprocessing protocol 1	Mosaic merging (Merge, Data Management, ArcGIS)	Mean predicted tidal levels obtained for 582 standard (tabulated) and secondary ports (calculated from the standard ports)
Preprocessing protocol 2	Interpolate to 1 m horizontal resolution (Aggregate, Spatial Analyst, ArcGIS)	
Preprocessing protocol 3	Gap interpolation (Nibble, Spatial Analyst, ArcGIS)	
Preprocessing protocol 4	Cropping to relevant area delimited by HAT (Polygon feature, ArcGIS)	
Preprocessing protocol 5	Slope maps acquisition (Slope, Data Analyst, ArcGIS)	
Preprocessing protocol 6	Convert to ASCII (Raster to ASCII, Conversion, ArcGIS)	
Processing protocol 1	Creek detection (Fig. 3, Phase 1)	Interpolation from the weighted mean of the surrounding ports’ values up to 30 km away
Processing protocol 2	Creek repair (Fig. 3, Phase 2)	
Processing protocol 3	Parameters extraction (Fig. 3, Phase 3)	
Outputs	Creek extent mask Creek order skeleton maps Creek morphological parameters	Interpolated mean tidal levels (HAT, MHWS, MHWN, MWS, MLWN, MLWS)

**Table 3:** Summary of data record (access: https://eprints.soton.ac.uk/434946/).

Name	Output Type	Provenance	Experimental manipulations	Content
SM1	MR information	Academic and grey literature, online database ABPmer OMReg	Qualitative data collation from various sources	Time embanked, Policy context, Site aims, Number of breaches, Initial creek system, Design details, References
SM2	Mean tidal levels	Admiralty Tide Table 2014	Tide data preprocessing protocol 1, processing protocol 1	HAT, MHWS, MHWN, MWS, MLWN, MLWS
SM3	XYZ area maps	Environment Agency	Lidar data preprocessing protocols 1–4, processing protocol 1	textfiles containing XYZ data that cover the area and topography of the whole managed realignment schemes
SM4	XYZ creek maps	Environment Agency	Lidar data preprocessing protocols 1–6, processing protocols 1–2	textfiles containing XYZ data that cover the area and topography of creek networks
SM5	XYC creek skeleton maps	Environment Agency	Lidar data preprocessing protocols 1–6, processing protocols 1–3	textfiles containing XYC data that give the skeletonized area and Reverse Strahler order of creek networks
SM6	Morphometric parameters and figures	Environment Agency, Admiralty Tide Table 2014	Lidar data preprocessing protocols 1–6, processing protocols 1–3	PDF file containing:
				-table of creek network morphometric parameters (Reverse Strahler order, Number of creeks per order, Total Length, Mean Length, Bifurcation Ratio, Sinuosity Ratio, Mean junction angle, Mean channel width, Mean channel depth, Mean cross-sectional area, A/D, W/D, Drainage density, Total Channel Length, Catchment area, Mean elevation above MWS, Overmarsh Path Length)
				-Figures of cross-sectional area evolution of the largest entry channel mouth for all MR sites and all available years.
				-Figures of MR creek morphometric parameters evolution per RS order over the years, plotted against the 95% spread of natural creek parameters.
				-Figures showing the evolution of the MR creek extent, unchanneled length and overmarsh path length over the years for all sites
				-Figures showing the elevation gains and losses of all MR sites between the first and the last year considered, correlated to initial site elevation and distance to creeks.
CHIROL CREEK EXTRACTION DEMO	Custom code used to generate the data	CHIROL-CREEK-ALGORITHM is a semi-automated coastal wetland creek parametrisation tool developed in 2018 by Clementine Chirol as part of her PhD at the University of Southampton, from 2014 to 2018, in collaboration with Jacobs. This tool uses a semi-automated elevation and slope thresholds method to detect a creek network, and gives as outputs an Excel table of morphometric characteristics and several figures. The scripts are run on MatLAB2015a.		

## Data Records

The database presented herein consists in a set of tables, figures and text files containing creek network morphological characteristics for each of the 10 MR sites. The data and the algorithm used to generate it are accessible via ePrints (https://eprints.soton.ac.uk/434946/)^44^ and via the Channel Coastal Observatory website (https://coastalmonitoring.org/ccoresources/education/chirol/). The complete list of data made available in the repository is as follows (summarised in Table 3):

Supporting Material SM1 is a table listing the initial conditions, design choices and available lidar datasets for each MR scheme considered (**Stage 1**). The table contains for each scheme: Implementation date, Latitude/Longitude, Catchment area, Time embanked (with references), Policy context, Site aims, Number of breaches (if MR), Initial creek system, Design details, Other references, available lidar data at 1 m resolution. The background information has been collected from various sources as listed in Table 1.

Supporting Material SM2 is a table listing the tidal data obtained for each site, including: Highest Astronomical Tide, Mean High Water Spring, Mean Low Water Spring, Mean Spring Tidal Range, Mean High Water Neap, Mean Low Water Neap and Mean Neap Tidal Range (**Stage 1**).

Supporting Material SM3 provides 52 text files containing XYZ data that cover the extent and elevation of UK managed realignment schemes, for all study sites and all available years, after implementing preprocessing protocols 1–4 (**Stage 2**).

Supporting Material SM4 provides 52 text files containing XYZ data that cover the extent and elevation of creek networks as detected by lidar and our developed algorithm for all study sites and all available years (**Stage 3**).

Supporting Material SM5 provides 52 text files containing XYC data that cover the skeletonized creek network area as detected by lidar and our developed algorithm for all study sites and all available years, and gives the Reverse Strahler order of each branch (**Stage 3**).

Supporting Material SM6 provides tables and supporting figures that describe the morphological evolution of creek networks in MR schemes following implementation (**Stage 3**). They cover a broad range of parameters to facilitate their reuse for future studies. These are concatenated in a PDF file, and numbered Appendix A to E:

- Appendix A is a table listing all results from the creek network morphometric analysis performed at 10 MR schemes in the UK, utilising the semi-automated creek parametrisation algorithm for each available lidar dataset.
- Appendix B consists of 10 figures showing the cross-sectional area evolution of the largest entry channel mouth for all MR sites and all available years.
- Appendix C consists of 10 figures showing the evolution of MR creek morphometric parameters per Reverse Strahler order over the years, plotted against the 95% spread of natural creek parameters.
- Appendix D consists of 10 figures showing the evolution of the MR creek extent, unchanneled length and overmarsh path length over the years for all sites
- Appendix E consists of 10 figures showing the elevation gains and losses of all MR sites between the first and the last year considered, and correlates them to the initial site elevation and distance to creeks to analyse creek forming processes. The effect of creek proximity on the creek network is evaluated up to 20 m away from the creeks to reduce the impact of site elevation or of multiple creek influence on the results. Positive elevation changes are correlated to the most recent creek extent and negative elevation changes to the initial creek extent.

Finally, CHIROL_CREEK_EXTRACTION_DEMO contains the code used to generate the dataset, in the form of a list of Matlab functions and scripts. The associated READ_ME file contains a utilisation guide detailing the order in which the three primary Matlab functions should be called.

## Technical Validation

The various validation tests described below were applied to the lidar datasets and to the creek algorithm results.

### Lidar data validation

The following quality control procedures were undertaken on the lidar datasets to justify carrying out a multi-annual monitoring of coastal wetlands using lidar:

1. Tests of the repeatability of lidar data collection over different years;
2. Investigations of the potential systematic bias caused by the presence of low-lying saltmarsh vegetation in the DSM and DTM; and
3. Comparison of the respective efficiency of the DSM and DTM at detecting the marsh surface

#### Repeatability of lidar

The repeatability of lidar data was tested for 5 different datasets collected near HOMW in 2007, 2009, 2010, 2011 and 2014. Elevation values were found using the nearest neighbour interpolation method along a low-lying unmoving feature, in this case a road (Appendix A1). The largest vertical repeatability error estimated from the mean standard deviation for the five points is +/−0.06 m, which is less than the 0.15 m vertical resolution of lidar.

#### Effect of vegetation

**Fig. 4:**
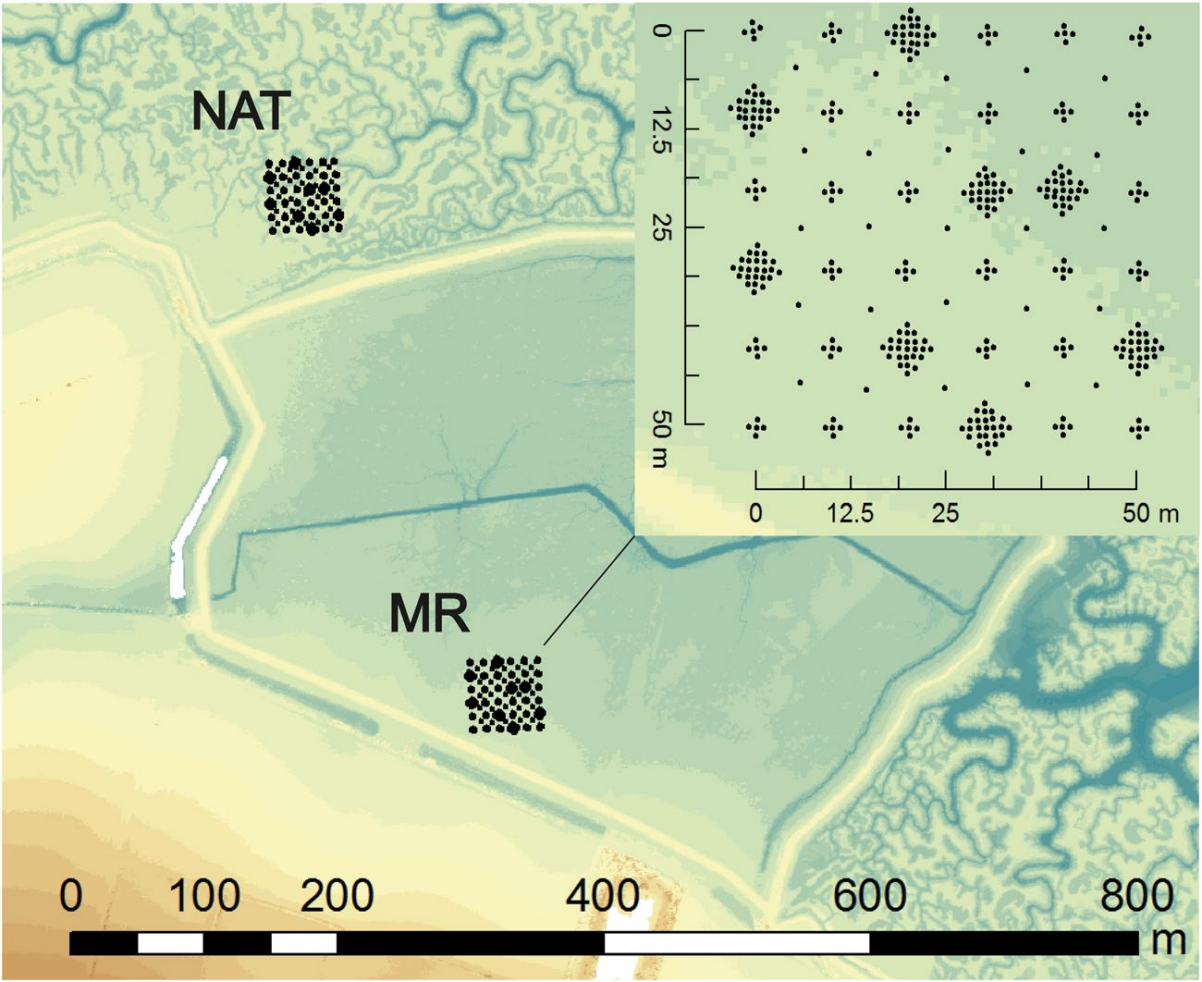
Layout of RTK-GPS points used for validation of lidar data. The RTK GPS points were collected in August 2015 in a natural marsh (NAT) and in the Tollesbury MR schemes (MR), overlain over lidar DSM collected in February 2015. RTK GPS dataset curtesy of Peter Lawrence.

The effects of saltmarsh vegetation can lead to an overestimation of the marsh surface elevation by lidar and a systematic bias in the dataset. This effect needs to be quantified. Also, the capacity of the EA proprietary vegetation removal algorithms at minimising this bias needs investigating. Real-Time Kinematic (RTK) GPS data were collected with an accuracy <2 cm and a precision <1.5 cm in August 2015 in a MR scheme at Tollesbury and in an adjacent natural mature saltmarsh (Fig. 4). The marsh surface elevation data relative to ODm were collected across a 50 × 50 m section following the systematic grid sampling method described by Brooks *et al*.^45^ and expanded by Lawrence (2018)^46^. 36 focal sampling points are selected every 10 m, with 4 additional sampling points at 1 m N, S, E and W of each focal points. For 8 randomly selected focal points, 20 additional points are taken at 2 and 3 m N, S, E and W of each focal point. Finally, 25 additional points are selected within the grid at 5 m N, S, E and W from the focal points.

The method is used to study ground heterogeneity over length scales ranging from 1 to 80 m, and is well applicable to lidar ground-truthing. The closest in time lidar data available are from the 22^nd^ February 2015. Contrary to the GPS data lidar may detect clumps of vegetation. Some marsh accretion may have occurred between February and August 2015; however, with accretion rates in Tollesbury MR of 2.3 cm/yr to 2.89 cm/yr as estimated by the present and a previous study^47^, the difference is unlikely to be detected by RTK GPS or lidar. Most of the differences between the two datasets should thus be due to the vegetation present in February 2015.

**Fig. 5:**
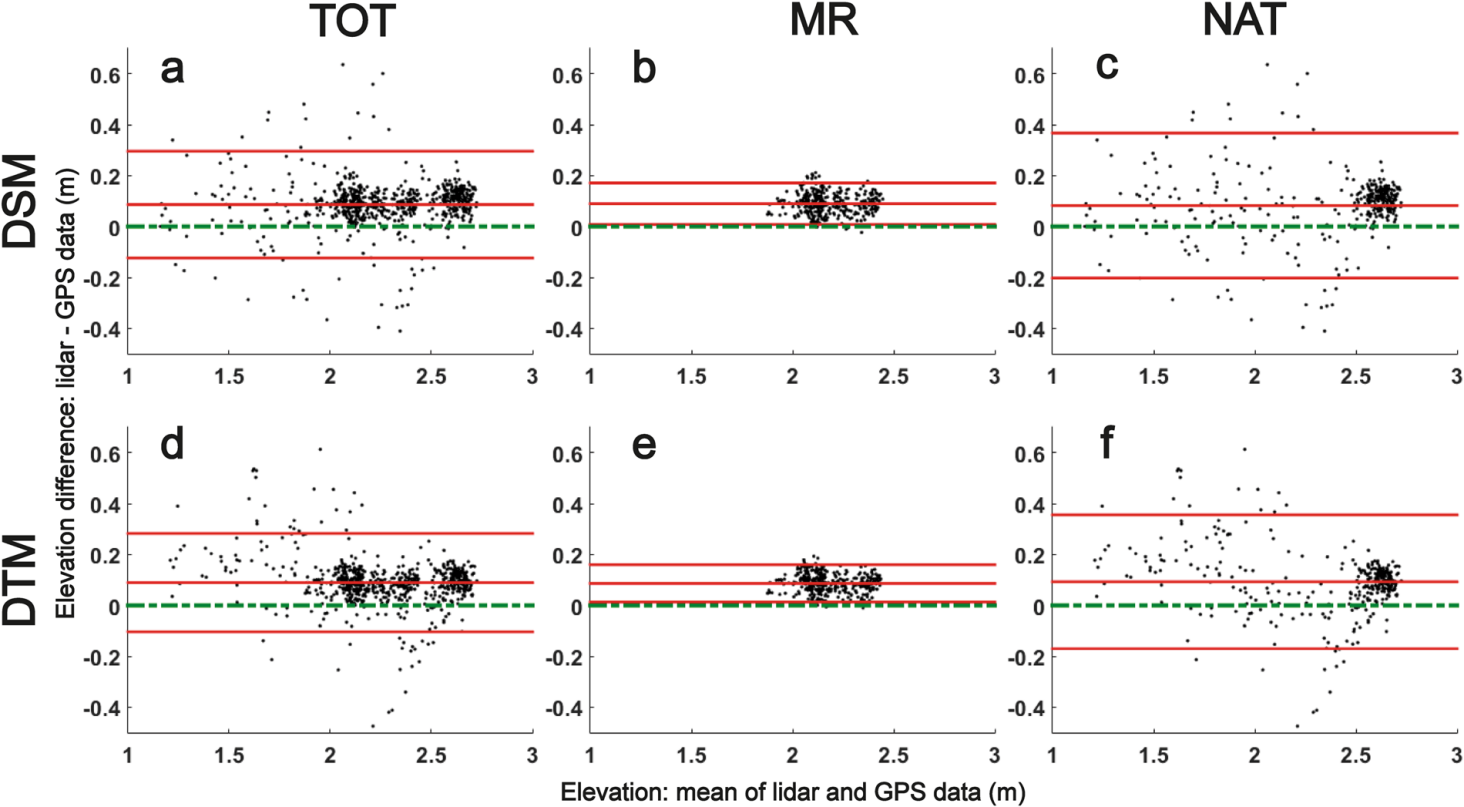
Pairwise difference between RTK GPS and lidar data in saltmarshes. The differences between lidar (**a–c**: DSM and **d–f**: DTM) and RTK-GPS data are investigated at a natural (NAT) and at an artificial (MR) marsh in Tollesbury. The sum of both locations is marked under TOT. The three red lines show the limits of agreement (2x standard deviation) and the mean value of the differences. The dashed green line shows the ideal mean difference if there is no bias between the two methods.

Most data points (97% using the DSM and 96% using the DTM) fall within the limit of agreement (Fig. 5). A positive systematic bias of 0.09 m was found between the lidar and GPS data, for both the DSM and the DTM: lidar tends to overestimate the elevation values by 0.09 m. This value fits with the expected elevation of dense saltmarsh canopy in the UK^48^. The persistence of this positive bias in the DTM shows that the EA vegetation removal algorithms fail to systematically remove the vegetation cover. This is expected when the vegetation cover is lower than the vertical resolution of lidar; however previous studies have found lidar DTM to record dense clumps of reeds as high as 2 m^49^. The Root Mean Square Error of the lidar versus GPS elevation data is higher for the natural saltmarsh (0.13 m) than for the MR scheme (0.07 m), probably due to the higher ground heterogeneity^45^ and higher vegetation diversity found in natural saltmarshes^30^.

Finally, some fluctuation can be expected in the mean vegetation cover between summer and winter^50^. This should be visible as fluctuations in the mean marsh elevation data from year to year. If interannual fluctuations in the mean marsh elevation data are small, and if the elevation changes are linear, then it can be assumed that the changes are due to accretion rather than vegetation growth and die-back. Elevation changes data as detected by lidar should also be confronted with field monitoring data of accretion rates when available in the literature.

#### DSM/DTM comparison

Compared to the uncorrected DSM, the EA proprietary DTM generation algorithms aim to remove first return points corresponding to vegetation or standing water, and to replace them with points of latter arrival corresponding to the ground. In order to explore the efficiency of those algorithms, the DTM was subtracted from the raw DSM in HOMW, were trees were present in 2009, for 5 different years. Most of the differences between the models occur along and inside the creek network, where the elevation is higher in the DSM than in the DTM. Some of the larger positive values correspond to trees, isolated or in a line along one of the main channels. According to the standard deviation values, discrepancies of about 0.26 m can be expected between the DSM and the DTM.

However, overall the amount of correction differs greatly between years: it is suspected that the algorithm itself has been updated over time, but that the newest algorithm has not been systematically applied to the older datasets, leading to inconsistencies. Some tiles in the 2007 dataset also seem to have undergone no correction at all, showing that the algorithms are not always systematically applied, in accordance with previous findings^51^.

Uncertainties concerning the functioning and consistency of application of EA algorithms make the DTM datasets unreliable for monitoring the morphological evolution of coastal wetlands. Furthermore, since the DTM datasets do not remove the systematic bias caused by low saltmarsh vegetation as seen previously, this study uses the raw DSM. This approach assumes that the low vegetation cover characteristic of saltmarsh areas is unlikely to mask the creek network or significantly affect the detection of creek edges, as based on aerial photography and field observations, plants develop on the creek banks but rarely within the creeks themselves. This choice might lead to underestimation of the channel depth, if the laser is reflected by residual water within the creek during low tide. It could also lead to overestimation of the saltmarsh elevation if the vegetation cover is detected as the ground level, and could limit the monitoring of accretion rates when performing the MR evolution analysis over several years^52,53^.

Some open-source algorithms attempt to correct vegetation from lidar data, but 1) they rely on precise knowledge of the vegetation distribution on site, obtained through regular field surveying, which defeats the purpose of lidar as a quick and cheap monitoring method over large areas, and 2) the correction factors based on local dominant vegetation height creates unrealistic “steps” in the dataset, which may complicate the detection of creek networks^53^. Overall, vegetation removal has been a major issue in previous lidar-based saltmarsh monitoring studies ^52^ :even state of the art lidar sensors fail to penetrate the saltmarsh canopy^54^. This study provides an opportunity to estimate the efficiency of the uncorrected DSM at detecting evolving creek systems.

### Creek algorithm validation

**Fig. 6:**
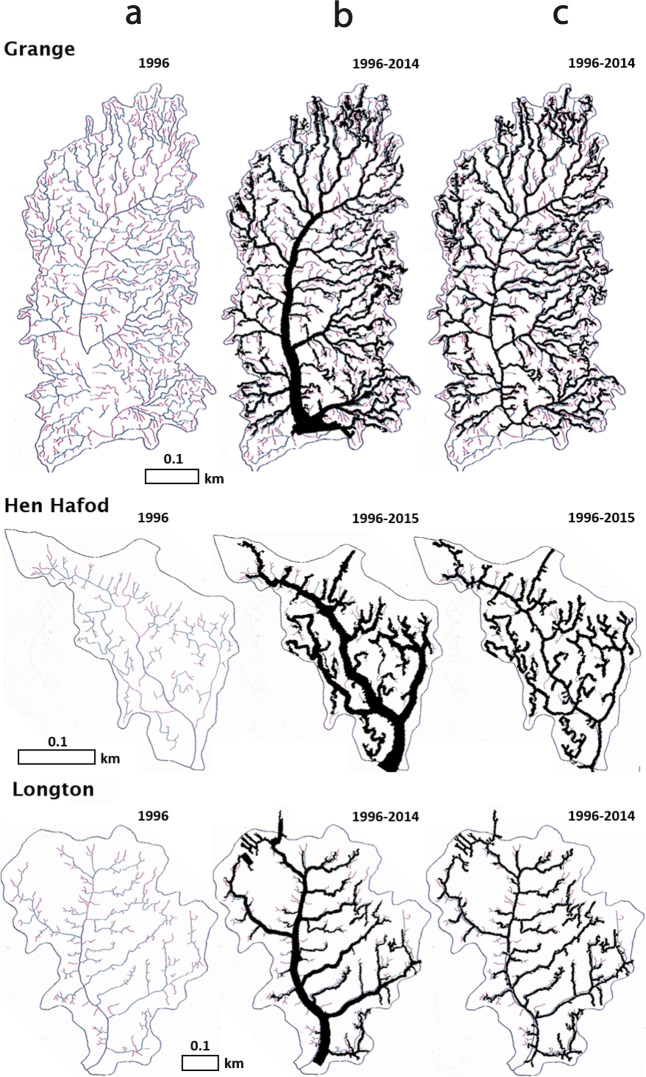
Creek area and skeleton extraction result comparison for three saltmarshes using manual and semi-automated mapping. The creek networks in the natural saltmarshes Grange, Hen Hafod and Longton were manually mapped by Steel in 1996^55^, then mapped semi-automatically using lidar data collected in 2014 and 2015. (**a**) Creek network skeleton manually extracted by Steel. (**b**) creek mask. (**c**) creek skeleton (**b** and **c** overlain over Steel’s manual extraction results).

In order to verify its accuracy, the creek parametrisation algorithm was tested on 13 mature natural British saltmarshes. Creek morphological parameters were obtained independently from manual mapping and field surveying^55^. We extracted creek parameters for those sites from recent lidar datasets (2014–2016) using the newly developed algorithm. Supposing that the natural saltmarshes are at equilibrium and that the effect of sea-level rise on marsh extent is negligible over the considered timescale, the algorithm should yield similar creek parameters. An example of comparison between the two creek mapping methods is given in Fig. 6, while the complete validation process is detailed in Chirol *et al*.^21^.

**Fig. 7:**
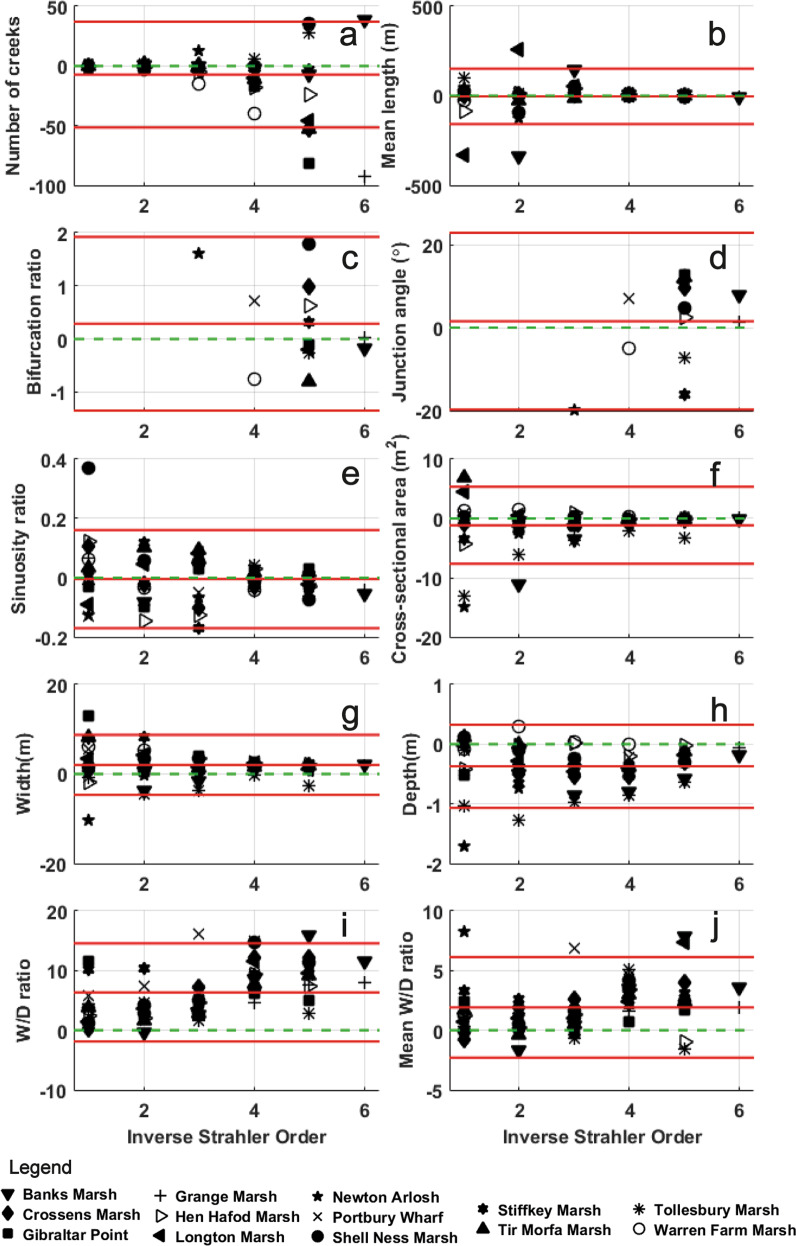
Morphological parameter differences for each reverse Strahler order (lidar extraction results minus Steel results^55^). (**a**) number of creeks; (**b**) mean length; (**c**) bifurcation ratio; (**d**) junction angle; (**e**) sinuosity ratio; (**f**) cross-sectional area; (**g**) creek width; (**h**) creek depth; (**i**) width/depth ratio from top of creek; (**j**) area/depth^2^ ratio (mean width/depth ratio). Red lines show the limits of agreement (2* standard deviation) surrounding the mean value of the differences. The dashed green line shows the ideal mean difference of 0 if there is no bias between the two methods.

In order to assess the agreement between the two pairs of readings, detect outliers and visualise systematic biases, the differences were plotted on a Bland and Altman diagram (Fig. 7). Most of the differences between parameters lie within the limits of agreement of 2* standard deviation. The errors of omission in creek number compared with Steel’s results generally increase for the smaller creeks of higher Reverse Strahler Orders (Fig. 7a). No visible bias was found in the detection of mean creek length (Fig. 7b) and junction angle (Fig. 7d). Data from both methods were in good agreement for the creek numbers (negative mean difference of 7 creeks with the algorithm, Fig. 7a), the cross-sectional areas (mean negative difference of 1.1 m^2^, Fig. 7f) and the bifurcation ratio (mean positive difference of 0.3, Fig. 7c). The depth of channels measured using lidar were ~ 0.4 m shallower than Steel’s (1996) field validated results (Fig. 7h), probably due to the presence of residual water at the bottom of creeks: this is a limitation of using near infrared lidar data which cannot penetrate water^56^. Furthremore, creek width is overestimated when adjacent creeks are detected as one channel due to the resolution of the dataset, leading to a mean difference of 2 m (Fig. 7g). Width overestimation and depth underestimation leads to a width/depth ratio overestimation of 6.3 compared to Steel’s (1996) results (Fig. 7i). Even though the values fell within those expected of intertidal creek networks, between 5 and 34^43^, no correlation could be found between the width/depth ratio and the reverse Stralher order. However, in the case of the cross-sectional area and the mean width value (given by the area/depth), the depth underestimation had a much lower impact, and the results are close to Steel’s (1996)^55^, with higher values for first reverse Strahler order channels, and a positive mean difference of 1.9 for the mean width/depth ratio (Fig. 7j).

**Fig. 8:**
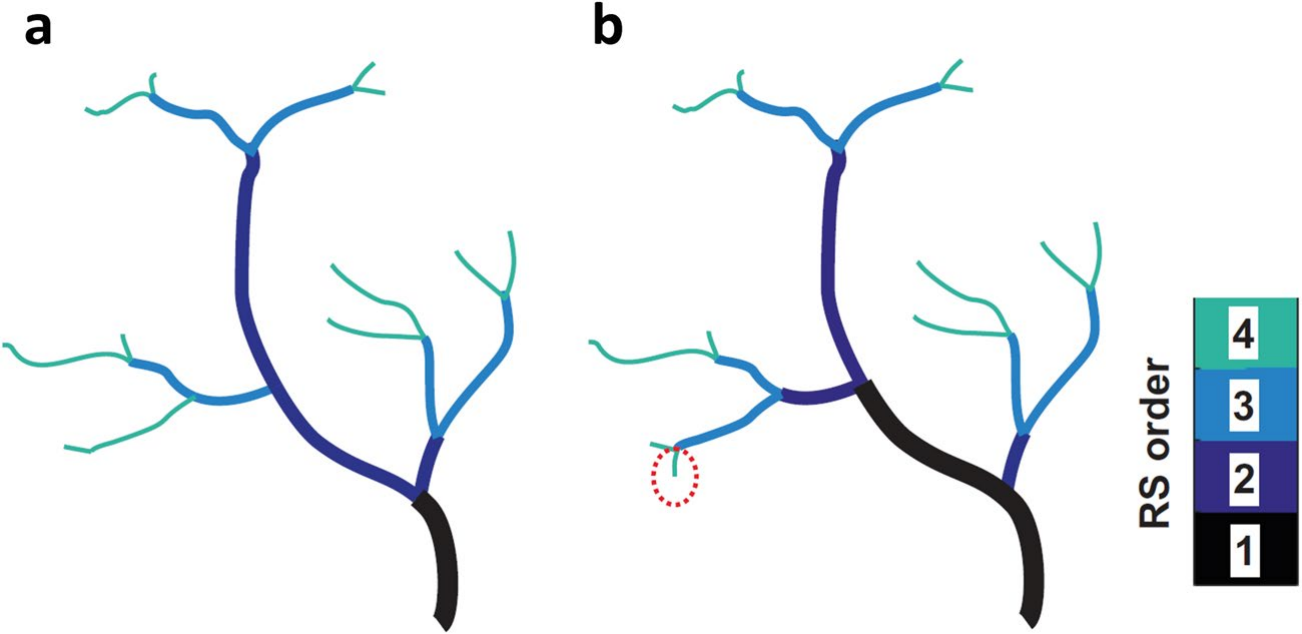
Illustration of how subtle differences in creek detection can significantly impact branching and creek order throughout the system (valid for both Strahler and Reverse Strahler ordering). Detection method **a** led to one 4^th^ Reverse Strahler (RS) order creek to be omitted (red dashed circle) compared with method **b**, with a knock-on effect on the rest of the creek system, notably the length of the large RS order 1 creek. The detected entry channel (RS order 1) is twice as long and sinuous in **b** than in **a**.

Outliers in creek length for the entry channels and largest tributaries (first and second reverse Strahler orders) can generally be explained by the differences in detected creek branching. Indeed, the bifurcation ratio varies between −1 and 2 depending on the creek extraction method chosen (Fig. 7c): those variations in detected branching change the distribution of creek orders, with a potential knock-on effect on the rest of the creek system (Fig. 8). A good example of this is the detected creek length at Longton marsh (Fig. 7b), where the first reverse Strahler order length is significantly underestimated while creek length is overestimated for the second order: subtle differences in the detection of high order creeks led to vastly different characteristics of the entry channel. However, this problem does not affect the characteristics of the whole system such as the total channel length, drainage density, total creek volume and OPL.

Overall, there is a good agreement between the field-validated creek morphological parameters and those extracted by the algorithm, showing that this method applied to 1 m horizontal and 0.15 m vertical resolution is adequate to capture the evolution of the majority of active creeks. The chosen resolution for the lidar data also means that the likelihood of detecting ephemeral creeks is low. Most of the differences are linked to limits in ground detection by lidar, due to the presence of remnant water within creeks or of low-lying dense vegetation. Small differences in creek detection can have a knock-on effect on creek ordering, but the general creek characteristics such as total channel length, volume and OPL remain unaffected. The uncertainty linked to creek detection can be quantified using the standard deviation of morphological parameter values when the elevation thresholds vary by +/− 0.15 m. This uncertainty was tested for Hesketh Out Marsh West (HOMW), one of the largest and more complex creek networks considered, using 4 lidar datasets taken between 2009 and 2014 (Table 4).

**Table 4:** Creek morphological parameters used in the study and data processing uncertainty mean calculated as the standard deviation of morphological parameters detected by the algorithm at Hesketh Out Marsh West (HOMW) when the elevation thresholds are changed by +/− 0.15 m.

Parameter (tested on HOMW 2009–2014)	Symbol	Mean standard deviation (% of mean value)
Mean elevation above MWS (m)	MWS	N/A
Drainage density (km/km^2^)	DD	0.5 (5%)
Overmarsh path length (m)	OPL	5.08 (11%)
Main channel length (m)	MCL	36.8 (3%)
Total channel length (m)	TCL	800 (5%)
Number of creeks (no unit)	NB	21.2 (9%)
Total mouth cross-sectional area (m^2^)	CSA	4.98 (4%)
Main channel mouth depth (m)	D	0.33 (12%)
Planform area (m^2^)	PA	3.49*10^4^ (20%)
Undermarsh tidal prism (creek volume) (m^3^)	TP	2.02*10^4^ (17%)
Sinuosity ratio (no unit)	SR	0.07 (6%)
Main channel gradient (°)	MCG	0.05 (4%)

## Data Availability

The custom code used to produce the dataset, written in Matlab R2015a, is available within the data repository via https://eprints.soton.ac.uk/434946/ (see CHIROL_CREEK_EXTRACTION_DEMO zip folder and utilisation guide README_Creek_Extraction_Algorithm). Readers can also refer a previously published methodology paper^21^ which details the algorithm’s functioning and validation process.
